# Efficacy and safety of PD‐1 inhibitor combined with antiangiogenic therapy for unresectable hepatocellular carcinoma: A multicenter retrospective study

**DOI:** 10.1002/cam4.4747

**Published:** 2022-04-10

**Authors:** Junlin Yao, Xudong Zhu, Zhiheng Wu, Qing Wei, Yibo Cai, Yu Zheng, Xinyu Hu, Hong Hu, Xiangyu Zhang, Hongming Pan, Xian Zhong, Weidong Han

**Affiliations:** ^1^ Department of Medical Oncology, Sir Run Run Shaw Hospital, College of Medicine Zhejiang University Hangzhou Zhejiang China; ^2^ Department of Medical Oncology, The First Affiliated Hospital, College of Medicine Zhejiang University Hangzhou Zhejiang China; ^3^ Department of Medical Oncology Cancer Hospital of the University of Chinese Academy of Sciences Zhejiang Cancer Hospital Hangzhou Zhejiang China; ^4^ Department of Colorectal Surgery Cancer Hospital of University of Chinese Academy of Sciences Zhejiang Cancer Hospital Hangzhou Zhejiang China; ^5^ Department of General Surgery, Sir Run Run Shaw Hospital, College of Medicine Zhejiang University Hangzhou Zhejiang China; ^6^ Department of Medical Oncology, The Second Affiliated Hospital, College of Medicine Zhejiang University Hangzhou Zhejiang China; ^7^ Present address: Shaoxing Shangyu Hospital of Traditional Chinese Medicine Shangyu Zhejiang China

**Keywords:** angiogenesis, hepatocellular carcinoma, immunology, prognosis, prognostic factor

## Abstract

**Background:**

Immunotherapy‐antiangiogenesis combination therapy has achieved excellent survival outcomes in hepatocellular carcinoma (HCC) in clinical trials. However, the combination therapy for HCC outside clinical trials is not well studied, and predictive factors are lacking. Here, we retrospectively analyzed the efficacy and safety of immunotherapy‐antiangiogenesis combination therapy in unresectable HCC patients in a real‐world setting.

**Methods:**

We conducted a four‐center, retrospective study of unresectable HCC patients who received the combination of programmed death 1 **(**PD‐1) inhibitor and antiangiogenic agent between April 2018 and July 2021 in China.

**Results:**

In total, 136 patients were enrolled in the cohort. The objective response rate (ORR) and disease control rate (DCR) were 38.0% and 81.8%, respectively. The median time to progression (TTP), progression‐free survival (PFS), and overall survival (OS) were 7.2, 7.3, and 19.6 months, respectively. The multivariate analysis indicated that ECOG performance status score (PS) 2 was a significantly independent negative factor of ORR. Moreover, ECOG PS 2, peritoneum metastasis and previous immunotherapy were found to be independent negative predictors of PFS. A shorter OS was associated with ECOG PS 2, peritoneum metastasis, the presence of previous immunotherapy, Child‐Pugh stage B, and high alpha‐fetoprotein (AFP) concentration. One hundred and twenty‐five patients (91.9%) reported adverse events (AEs) with any grade.

**Conclusion:**

We elucidated the efficacy and safety of immunotherapy‐antiangiogenesis combination therapy and identified potential predictors for response and survival in a real‐world cohort of patients with unresectable HCC.

## Graphical abstract

Immunotherapy‐antiangiogenesis is an efficacious strategy for unresectable HCC. We identified potential predictors for response and prognosis in HCC.

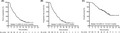

## 1 INTRODUCTION

Hepatocellular carcinoma (HCC) comprises 75%–85% of case of primary liver cancer, which is the sixth most prevalent cancer and the third leading cause of cancer death worldwide in 2020.^1^ The majority of HCC patients are diagnosed at an advantage stage or progress following surgery and locoregional therapy (LRT) initiation, systemic therapy is the appropriate option for these patients and the prognosis is usually poor.^2^

Vascular endothelial growth factor receptor (VEGFR)‐targeted tyrosine kinase inhibitor (TKI) sorafenib has been the standard systemic therapy for advanced HCC for a long time since 2007.^3^ Besides, many other TKIs have been approved to effectively target HCC and added as the first‐line (lenvatinib) or second‐line (regorafenib, apatinib, ramucirumab, and cabozantinib) systemic therapy for HCC since 2018.^4^, ^5^, ^6^, ^7^, ^8^ Furthermore, we are seeing an evolving landscape of immunotherapy toward HCC. Two programmed death 1 (PD‐1) inhibitors, pembrolizumab and nivolumab, have shown promising efficacy and acceptable safety in phase 2 KEYNOTE‐224 and CheckMate‐040 studies, respectively.^9^, ^10^ Whereas, the disappointing overall survival (OS) results in the phase 3 KEYNOTE‐240 and CheckMate‐459 studies frustrated administering PD‐1 inhibitor alone in advanced HCC.^11^, ^12^ Most current developments in combination strategy include anti‐cytotoxic T lymphocyte‐associated protein 4 (CTLA‐4) plus anti‐PD‐1 antibody (e.g., ipilimumab + nivolumab),^13^ TKI plus anti‐PD‐1 antibody (e.g., lenvatinib + pembrolizumab),^14^ and the combination of antibodies against PD‐ligand 1 (PD‐L1) and vascular endothelial growth factor (VEGF), namely atezolizumab plus bevacizumab.^15^

In detail, atezolizumab in conjunction with bevacizumab showed superior PFS (6.8 months vs. 4.3 months, *p <* 0.001) and OS (19.2 months vs. 13.4 months, *p* = 0.0009) compared to sorafenib in the phase 3 IMbrave 150 trial,^15^, ^16^ demonstrating the synergistic effects of PD‐1/PD‐L1 inhibitor and VEGF/VEGFR‐based antiangiogenic therapy. However, the response pattern to the combination therapy of PD‐1/PD‐L1 inhibitor and antiangiogenic agent varies among HCC patients, since it may derive from tumor heterogeneity, metastasis locations, and tumor microenvironment.^17^, ^18^, ^19^ Combined with LRT might be an option to optimize treatment strategy,^20^ and identification of a patient subset who could benefit from the combination therapy is also indispensable for clinical treatment practices.

In this real‐world cohort study, we retrospectively analyzed the efficacy and safety of PD‐1 inhibitor plus antiangiogenic therapy in unresectable HCC patients, and integrated clinical characteristics obtained from HCC patients in order to identify possible prognostic factors for response and prognosis.

## 2 MATERIALS AND METHODS

### 2.1 Patients

This was a retrospective study of patients with unresectable HCC who treated with PD‐1 inhibitor plus antiangiogenic agent from April 2018 to July 2021 across four centers in China: (1) Sir Run Run Shaw Hospital, Zhejiang University; (2) The First Affiliated Hospital, Zhejiang University; (3) The Second Affiliated Hospital, Zhejiang University; (4) Zhejiang Cancer Hospital, Cancer Hospital of the University of Chinese Academy of Sciences.

Patients were included based on the following specific criteria: (1) HCC diagnosis based on histology or imaging modality; (2) patients not available for surgery, radiation or ablation; (3) patients treated with or without LRT synchronously, including transcatheter arterial chemoembolization (TACE), ablation, radiation, or seed implantation; (4) at least one measurable tumor lesion as conformed by Modified Response Evaluation Criteria in Solid Tumors (mRECIST). Patients were excluded if they presented (1) combined therapy for only one cycle; (2) no available follow‐up data; (3) no available data for baseline assessment and response assessment; (4) a second primary tumor in the recent 5 years; (5) pathologic finding was hepatocellular carcinoma with sarcoma or hepatic neuroendocrine carcinoma.

In total, data of 81 patients were excluded, the remaining 136 patients met the enrollment criteria and were enrolled for analysis. All data, including treatment strategy, laboratory results, and radiological assessments were collected from patients' electronic medical records. The study was performed in accordance with the Declaration of Helsinki and was approved by the Medical Ethics Committee of the four participating hospitals.

### 2.2 Treatment procedure

The combination strategy was determined based on previous treatment strategy, individual characteristics, patient willings, and economic consideration. Six available PD‐1 inhibitors were sintilimab, toripalimab, camrelizumab, pembrolizumab, nivolumab, and tislelizumab, which were administrated intravenously according to the following doses: sintilimab 200 mg, toripalimab 240 mg, pembrolizumab 200 mg, or tislelizumab 200 mg every 3 weeks, camrelizumab 200 mg, or nivolumab 3 mg/kg every 2 weeks. Simultaneously, patient received antiangiogenic therapy, including lenvatinib, sorafenib, regorafenib, apatinib, or bevacizumab, which was administered orally except bevacizumab. Lenvatinib was given 8 mg/day (body weight <60 kg) or 12 mg/day (body weight ≥60 kg). The initial dose of sorafenib was 400 mg/day and increased to 800 mg/day if tolerated. Regorafenib was given 80 mg/day from week 1 to 3 of every 4‐week cycle. Patients received apatinib at a dose of 250 mg daily or bevacizumab 7.5 mg/kg every 3 weeks intravenously.

According to tumor burdens and goal of treatment, selected patients received concomitant LRT for at least one‐time within 1 month before or after the combined systemic therapy, which including TACE, ablation, radiation and/or seed implantation. Patients who experienced serious treatment‐related adverse events (TRAEs) would have dose delay, dose reduction, treatment interruption, or discontinuation based on the grade of toxicity. Patients with active hepatitis B virus (HBV) infection received antiviral treatment synchronously.

### 2.3 Assessments

Radiological data were collected based on dynamic computed tomography (CT) and/or magnetic resonance imaging (MRI) at baseline and every 8–12 weeks thereafter. Tumor responses were evaluated according to mRECIST:^21^ (1) complete response (CR) as the complete disappearance of all target lesions in enhanced arterial phase; (2) partial response (PR) as a ≥30% decrease of the diameter of the target lesions in the arterial phase; (3) stable disease (SD) as between a 30% decrease and a 20% increase of the diameter of the target lesion; (4) progressive disease (PD) as ≥20% increase of the diameter of the target lesions (enhanced imaging in the arterial phase), or new lesions development.

ORR was determined as the sum of CR and PR. Disease control rate (DCR) was defined as the percentages of CR, PR, and SD. PFS referred to the time interval from treatment initiation to progression or death from any cause. Time to tumor progression (TTP) was determined as the time from the initial dose to progression confirmed by radiology. Overall survival (OS) was calculated from the start of the combination treatment until death. TRAEs were adverse events (AEs) that associated with PD‐1 inhibitor and antiangiogenic agent rather than LRT, which were collected according to the Common Terminology Criteria for Adverse Events, v 5.0.

### 2.4 Statistical analysis

Categorical variables in clinical characteristics were statistically analyzed by Pearson's X^2^ test or Fisher's exact test. The PFS and OS were estimated by Kaplan–Meier method, univariate analysis was preformed using Logrank test, all covariates with *p <* 0.05 in univariate analyses were then performed in a multivariate analysis using Cox proportional hazards regression model. The hazard ratio (HR) and confidence interval (CI) were calculated. Two‐sides *p* value <0.05 was considered statistically significance. Statistical analyses were conducted using IBM SPSS version 23 and GraphPad Prism version 8.00.

## 3 RESULTS

### 3.1 Baseline characteristics and therapeutic strategies

One hundred and thirty‐six unresectable, locally advanced or metastatic HCC patients who have received PD‐1 inhibitor plus antiangiogenic agent with or without additional LRTs were included in the retrospective cohort study (Figure S1), with the median follow‐up of 14.2 ± 6.4 months by the time of data lock (Aug 01, 2021).

The baseline patient characteristics are listed in Table 1. In brief, patients were predominantly male (*n* = 115, 84.6%) with the median age of 58 (range 14–84 years), 78 patients (57.4%) had an Eastern Cooperative Oncology Group performance status score (ECOG PS) of 0 or 1, 54 patients (39.7%) had a history of alcohol use. The majority of patients were positive for HBV infection (91.2%), and had liver cirrhosis (79.4%) with an AFP concentration below 400 IU/ml (53.7%). A total of 80.1% of patients were Child‐Pugh stage A and 91.2% of patients had Barcelona Clinic Liver Cancer (BCLC) stage C disease. One hundred and one patients (74.3%) had extrahepatic disease, lymph node (44.9%) was the most frequent site for metastasis, followed by lung (38.2%), peritoneum (8.8%), bone (8.1%), adrenal gland (5.1%), and intra‐abdominal implantation (5.1%). Additionally, half of patients (50.0%) had macrovascular invasion. Prior therapies were diverse, 67 (49.3%), 92 (67.6%), 14 (10.3%), and 47 (34.6%) patients received prior surgery, LRT, immunotherapy, and antiangiogenic therapy, respectively, and 56 patients (41.2%) received previous systemic treatment as first‐line or subsequent‐line therapy.

**TABLE 1:** Baseline characteristics

Characteristics and therapeutic strategies	*n* (%)
Ages (years)	
Median (range)	58 (14–84)
≥60	62 (45.6)
Sex	
Male	115 (84.6)
Female	21 (15.4)
ECOG performance status	
0–1	78 (57.4)
2	58 (42.6)
Alcohol use	
Current or previous	54 (39.7)
Never	82 (60.3)
Metastasis present	
Extrahepatic disease	101 (74.3)
Lung	52 (38.2)
Lymph nodes	61 (44.9)
Bone	11 (8.1)
Peritoneum	12 (8.8)
Intra‐abdominal implantation	7 (5.1)
Adrenal gland	7 (5.1)
Child‐Pugh stage	
A	109 (80.1)
B	27 (19.9)
BCLC stage	
B	12 (8.8)
C	124 (91.2)
Alpha‐Fetoprotein	
<400 (IU/ml)	73 (53.7)
≥400 (IU/ml)	63 (46.3)
Macrovascular invasion	68 (50.0)
Viral status	
Uninfected	12 (8.8)
Hepatitis B	124 (91.2)
Hepatitis C	0 (0)
Liver cirrhosis	108 (79.4)
Prior therapies	
Surgery	67 (49.3)
LRT^a^	92 (67.6)
Immunotherapy	14 (10.3)
Antiangiogenic therapy	47 (34.6)
Previous systemic treatment line	
0	80 (58.8)
≥1	56 (41.2)
With additional LRT^a^	63 (46.3)

Abbreviations: BCLC, Barcelona Clinic Liver Cancer; ECOG, Eastern Cooperative Oncology Group; LRT, locoregional therapy; TACE, transcatheter arterial chemoembolization.

^a^ LRT includes TACE, ablation, radiation, or seed implantation.

In the cohort, patients were given different combination strategy of PD‐1 inhibitors plus antiangiogenic agents (Table S1). The most frequently used PD‐1 inhibitor was sintilimab (41.9%), followed by toripalimab (25.0%), camrelizumab (22.1%), pembrolizumab (5.1%), nivolumab (3.7%), and tislelizumab (2.2%). Simultaneously, patients were treated with lenvatinib (41.9%), sorafenib (33.1%), regorafenib (11.8%), apatinib (11.0%), or bevacizumab (2.2%), which was mainly target VEGF/VEGFR‐driven angiogenic pathway. Meanwhile, a total of 63 patients (46.3%) received additional LRT during the systemic therapy, including TACE, ablation, radiation and/or seed implantation, over half of them (54.0%) had TACE therapy (Table S2).

### 3.2 Tumor response and potential predictors

Among 136 patients, 15 cases were not available for best response assessment, five patients died before the first image evaluation, 10 patients lost follow‐up image data, and remaining 121 patients had at least one available image for tumor response assessment. Complete radiographic response occurred in three (2.5%) locally advanced HCC patients with or without portal vein tumor thrombus. Two of them treated with additional TACE reached CR in 1.3 to 4.4 months, another without LRT had CR in 8.9 months. Forty‐three patients achieved PR, resulting in an ORR of 38.0%. Fifty‐three participants (43.8%) had SD and 22 participants (18.2%) had PD. The DCR was 81.8%. The best reduction from baseline in tumor measurement is shown in Figure S2.

Among the evaluated clinical characteristics, only ECOG PS was significantly associated with objective response to the combination therapy, results showed that the ORR was 32.2% in ECOG PS 0–1 group, while only 5.8% in ECOG PS 2 group (Fisher's exact test, *p <* 0.001) (Table 2). Other evaluated clinical characteristics, including age, sex, the history of alcohol use, site of metastasis, Child‐Pugh stage, BCLC stage, AFP concentration, macrovascular invasion, HBV infection, liver cirrhosis, and prior treatment, did not significantly influence the efficacy of PD‐1 inhibitor plus antiangiogenic agent toward HCC patients (Table 2).

**TABLE 2:** Relationship between patient clinical characteristics and treatment response

Characteristics	OR (95% CI)	*p* value
Age (<60 years vs. ≥60 years)	0.9 (0.4–1.9)	0.771
Sex (male vs. female)	0.7 (0.3–2.0)	0.543
ECOG performance status (0–1 vs. 2)	7.5 (3.0–18.9)	<0.001
Alcohol use (current or previous vs. never)	2.1 (1.0–4.6)	0.061
Lung metastasis (yes vs. no)	1.5 (0.7–3.1)	0.316
Lymph nodes metastasis (yes vs. no)	0.8 (0.4–1.8)	0.641
Bone metastasis (yes vs. no)	1.0 (0.3–3.9)	1.000
Peritoneum metastasis (yes vs. no)	1.0 (0.3–3.9)	1.000
Intra‐abdominal implantation (yes vs. no)	1.6 (0.3–8.5)	0.897
Adrenal gland metastasis (yes vs. no)	0.8 (0.2–3.8)	1.000
Child‐Pugh stage (A vs. B)	2.8 (1.0–8.1)	0.053
BCLC stage (B vs. C)	1.1 (0.3–4.1)	1.000
Alpha‐Fetoprotein (<400 IU/ml vs. ≥400 IU/ml)	0.7 (0.3–1.5)	0.332
Macrovascular invasion (yes vs. no)	0.8 (0.4–1.6)	0.498
Viral status (hepatitis B vs. uninfected)	1.4 (0.4–4.9)	0.836
Liver cirrhosis (yes vs. no)	1.7 (0.7–4.1)	0.282
Previous immunotherapy (yes vs. no)	2.0 (0.5–7.6)	0.506
Previous antiangiogenic therapy (yes vs. no)	1.7 (0.8–3.7)	0.190
Treatment systemic lines (0 vs. ≥1)	1.9 (0.9–4.1)	0.096
Previous surgery (yes vs. no)	0.9 (0.4–1.8)	0.694

Abbreviations: BCLC, Barcelona Clinic Liver Cancer; CI, confidence interval; ECOG, Eastern Cooperative Oncology Group; OR, odds ratio; vs., versus.

### 3.3 PFS, TTP, and clinical prognostic factors

**FIGURE 1:**
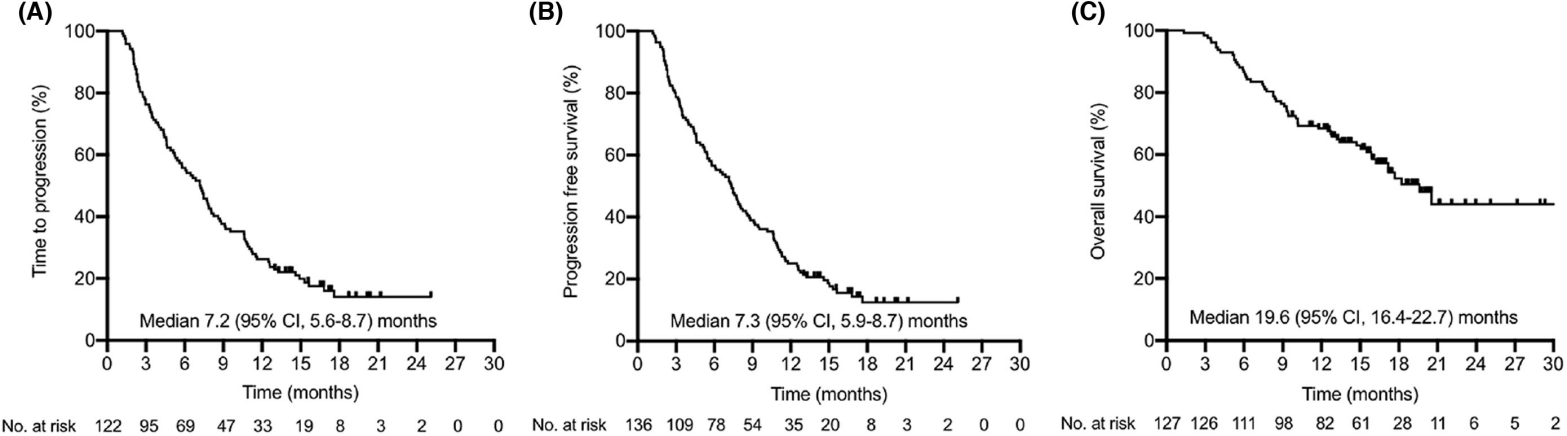
Kaplan–Meier estimates of TTP, PFS, and OS. (A) The median TTP was 7.2 months. (B) The median PFS was 7.3 months. (C) The median OS was 19.6 months. CI, confidence interval; OS, overall survival; PFS, progression‐free survival; TTP, time to progression

In the cohort, 121 cases were available for best response assessment, median time to progression (TTP) was 7.2 (95% CI, 5.6–8.7) months (Figure 1A), median PFS was 7.3 (95% CI, 5.9–8.7) months for all patients (Figure 1B). We then conducted PFS analyses of patients stratified by the evaluated clinical characteristics, most of the characteristics did not significantly influence PFS, while factors, including ECOG PS, lung metastasis, peritoneum metastasis, previous immunotherapy, and Child‐Pugh stage, were significantly associated with PFS by univariate analysis. Subsequently, the five significant factors were analyzed by Cox proportional hazards regression analysis, multivariate analysis identified independent predictors for PFS were ECOG PS, peritoneum metastasis and previous immunotherapy (Table 3). In detail, the median PFS of patients with ECOG PS 2 was significantly shorter than that of patients with ECOG PS 0–1 (3.5 vs. 9.2 months, *p* = 0.002), patients with peritoneum metastasis had a shorter PFS than those without peritoneum metastasis (3.2 vs. 7.5 months, *p* = 0.008). Moreover, patients who received prior immunotherapy had a shorter PFS than those who did not receive prior immunotherapy (3.9 vs. 7.6 months, *p* = 0.001).

**TABLE 3:** Univariate and multivariate analyses of the effects of clinical characteristics on PFS and OS

Characteristics	PFS				OS			
	Univariate analysis		Multivariate analysis		Univariate analysis		Multivariate analysis	
	HR (95% CI)	*p* value	HR (95% CI)	*p* value	HR (95% CI)	*p* value	HR (95% CI)	*p* value
Age (< 60 years vs. ≥ 60 years)	0.9 (0.6–1.3)	0.505	–	–	1.2 (0.7–2.0)	0.541	–	–
Sex (male vs. female)	1.2 (0.7–2.1)	0.453	–	–	1.2 (0.6–2.3)	0.676	–	–
ECOG performance status (0–1 vs. 2)	2.3 (1.5–3.4)	<0.001	1.9 (1.3–3.0)	0.002	3.0 (1.7–5.2)	<0.001	2.4 (1.3–4.5)	0.005
Alcohol use (current or previous vs. never)	1.1 (0.8–1.7)	0.476	–	–	1.0 (0.6–1.7)	0.948	–	–
Lung metastasis (yes vs. no)	1.5 (1.0–2.2)	0.049	1.2 (0.8–1.9)	0.298	2.0 (1.2–3.5)	0.007	1.2 (0.7–2.3)	0.429
Lymph nodes metastasis (yes vs. no)	1.0 (0.7–1.4)	0.861	–	–	1.3 (0.8–2.3)	0.262	–	–
Bone metastasis (yes vs. no)	1.4 (0.7–3.0)	0.285	–	–	1.3 (0.4–3.8)	0.665	–	–
Peritoneum metastasis (yes vs. no)	2.0 (0.9–4.6)	0.024	2.4 (1.3–4.5)	0.008	2.8 (1.0–8.1)	0.003	3.0 (1.4–6.7)	0.007
Intra‐abdominal implantation (yes vs. no)	0.9 (0.4–1.9)	0.760	–	–	0.6 (0.2–2.0)	0.509	–	–
Adrenal gland metastasis (yes vs. no)	1.4 (0.5–3.8)	0.376	–	–	1.8 (0.6–5.8)	0.205	–	–
Child‐Pugh stage (A vs. B)	1.9 (1.1–3.2)	0.004	1.5 (0.9–2.4)	0.116	3.3 (1.5–7.4)	<0.001	2.6 (1.3–4.9)	0.005
BCLC stage (B vs. C)	1.2 (0.7–2.2)	0.474	–	–	3.6 (1.6–8.1)	0.056	–	–
Alpha‐Fetoprotein, IU/ml (<400 vs. ≥400)	1.1 (0.7–1.6)	0.641	–	–	2.1 (1.2–3.6)	0.004	2.0 (1.1–3.4)	0.021
Macrovascular invasion (yes vs. no)	0.7 (0.5–1.0)	0.064	–	–	1.9 (1.1–3.2)	0.019	1.7 (0.9–3.1)	0.079
Viral status (hepatitis B vs. uninfected)	1.5 (0.8–2.8)	0.240	–	–	0.7 (0.3–1.9)	0.465	–	–
Liver cirrhosis (yes vs. no)	1.2 (0.8–1.9)	0.399	–	–	0.7 (0.3–1.3)	0.164	–	–
Previous immunotherapy (yes vs. no)	2.0 (1.0–4.3)	0.011	2.6 (1.4–4.5)	0.001	3.0 (1.1–8.5)	<0.001	3.2 (1.5–6.6)	0.002
Previous antiangiogenic therapy (yes vs. no)	1.0 (0.7–1.5)	0.784	–	–	1.1 (0.6–1.9)	0.746	–	–
Treatment systemic lines (0 vs. ≥1)	1.3 (0.9–1.9)	0.164	–	–	1.3 (0.7–2.1)	0.395	–	–
Previous surgery (yes vs. no)	0.8 (0.5–1.1)	0.142	–	–	0.8 (0.5–1.3)	0.311	–	–

Abbreviations: BCLC, Barcelona Clinic Liver Cancer; CI, confidence interval; ECOG, Eastern Cooperative Oncology Group; HR, hazard ratio; OS, overall survival; PFS, progression‐free survival; vs., versus.

### 3.4 OS and clinical prognostic factors

Of the 136 enrolled patients, 127 cases were available for OS assessment, the median OS was 19.6 (95% CI, 16.4–22.7) months (Figure 1C), 1‐year survival rate and 2‐year survival rate were 65% and 5%, respectively. The univariate and multivariate analyses were performed to identify prognostic factors associated with survival. Factors including ECOG PS, lung metastasis, peritoneum metastasis, previous immunotherapy, Child‐Pugh stage, AFP concentration, and macrovascular invasion were significantly associated with OS by univariate analysis. Of these factors, multivariate analyses verified that only poor ECOG PS (10.0 months vs. not reached [NR], *p* = 0.005), the present of peritoneum metastasis (12.5 months vs. NR, *p* = 0.007), previous immunotherapy (7.5 months vs. NR, *p* = 0.002), Child‐Pugh stage B (8.3 months vs. NR, *p* = 0.005), and high AFP concentration (15.5 months vs. NR, *p* = 0.021) were independent predictors for OS (Table 3).

### 3.5 Relation of treatment strategy and efficacy

Six available PD‐1 inhibitors and five antiangiogenic agents were applied in our study, the combination treatment strategy was individualized therapy. Considering the high cost of bevacizumab and the clinical practice in the real world,^22^ bevacizumab 7.5 mg/kg rather than 15 mg/kg was performed in HCC patients in our study. The relative dose intensity (RDI) and baseline characteristics of each antiangiogenic agent have been showed in Tables S3 and S4, respectively. Data showed that the type of antiangiogenic agent was associated with tumor response (Table S5), patients treated with lenvatinib had the highest ORR (50.0%), followed by those treated with sorafenib (30.4%), apatinib (10.9%), regorafenib (4.3%), and bevacizumab (4.3%). However, PD‐1 inhibitor type did not significantly affect tumor response, and type of both antiangiogenic agent and PD‐1 inhibitor was not a potential predictor for PFS and OS (Table S5).

In our study, 63 (46.3%) patients received additional LRTs combined with systemic therapy (Table S2), the ORR (52.2% vs. 47.8%, *p* = 0.31), and median PFS (7.3 vs. 7.5 months, *p* = 0.68) in LRTs group were comparable to those in non‐LRTs group, while patients in LRTs group had a longer OS than those in non‐LRTs group (NR vs. 16.2 months, *p* = 0.08), but had no significant differences (Table S5).

### 3.6 Adverse events

One hundred and twenty‐five patients (91.9%) experienced at least one TRAE (Table 4). Most common AEs were hypertransaminases (33.1%), thrombocytopenia (19.1%), hypertension (18.4%), leukopenia (18.4%), and hyperbilirubinemia (17.6%). Grade 3 and 4 AEs occurred in 38 (27.9%) patients while receiving treatment, the three most common ≥3 grade AEs were hyperbilirubinemia (5.9%), gastrointestinal bleeding (5.1%), and thrombocytopenia (4.4%). TRAE‐induced dose delay, dose reduction, treatment interruption, or discontinuation was required in 26 patients (19.1%). No patient died for TRAE in the cohort.

**TABLE 4:** TRAEs according to category and grade

	Total		PD‐1 inhibitors		Antiangiogenic agents	
	Any grade	Grade ≥3	Any grade	Grade ≥3	Any grade	Grade ≥3
TRAE	125 (91.9)	38 (27.9)	97 (71.3)	25 (18.4)	115 (84.6)	17 (12.5)
Specific TRAE						
Hypertransaminases	45 (33.1)	3 (2.2)	40 (29.4)	3 (2.2)	5 (3.7)	0
Thrombocytopenia	26 (19.1)	6 (4.4)	24 (17.6)	5 (3.7)	2 (1.5)	1 (0.7)
Hypertension	25 (18.4)	2 (1.5)	0	0	25 (18.4)	2 (1.5)
Leukopenia	25 (18.4)	5 (3.7)	25 (18.4)	5 (3.7)	0	0
Hyperbilirubinemia	24 (17.6)	8 (5.9)	21 (15.4)	7 (5.1)	6 (4.4)	1 (0.7)
Loss of appetite	21 (15.4)	0	21 (15.4)	0	21 (15.4)	0
Rash	19 (14.0)	2 (1.5)	14 (10.3)	2 (1.5)	5 (3.7)	0
Hypothyroidism	18 (13.2)	1	18 (13.2)	1 (0.7)	0	0
Anemia	17 (12.5)	0	17 (12.5)	0	0	0
Fatigue	16 (11.8)	0	16 (11.8)	0	16 (11.8)	0
Hand‐foot syndrome	15 (11.0)	4 (2.9)	0	0	15 (11.0)	4 (2.9)
Abdominal bloating	13 (9.6)	0	0	0	13 (9.6)	0
Proteinuria	12 (8.8)	2 (1.5)	0	0	12 (8.8)	2 (1.5)
Diarrhea	11 (8.1)	0	0	0	11 (8.1)	0
Gastrointestinal bleeding	9 (6.6)	7 (5.1)	0	0	9 (6.6)	7 (5.1)
Nausea/vomiting	10 (7.4)	1 (0.7)	0	0	10 (7.4)	1 (0.7)
Pruritus	5 (3.7)	0	5 (3.7)	0	0	0
Lymphopenia	3 (2.2)	0	3 (2.2)	0	0	0
Interstitial pneumonia	3 (2.2)	0	3 (2.2)	0	0	0
Hypophysitis	2 (1.5)	1 (0.7)	2 (1.5)	1 (0.7)	0	0
Hyperthyroidism	2 (1.5)	0	2 (1.5)	0	0	0
Oral mucositis	2 (1.5)	1 (0.7)	0	0	2 (1.5)	1 (0.7)
Elevated creatinine	2 (1.5)	0	0	0	2 (1.5)	0
Hemangioma	1 (0.7)	0	1 (0.7)	0	0	0
Thyroiditis	1 (0.7)	1 (0.7)	1 (0.7)	1 (0.7)	0	0

*Note*: Data presented as *n* (%).

Abbreviations: PD‐1, programmed death 1; TRAEs, treatment‐related adverse events.

## 4 DISCUSSION

Combination therapy by synergistic antitumor effects is suggested as an efficacious strategy for many cancers. The strategy using PD‐1/PD‐L1 inhibitor combined with antiangiogenic therapy has been successfully applied in HCC.^15^, ^23^ Herein, we retrospectively analyzed the efficacy and safety of PD‐1 inhibitor combined with antiangiogenic agent for unresectable HCC in the real world. The ORR of PD‐1 inhibitor plus antiangiogenic agent was 38.0% in our study, which was similar as the ORR (24%–46%) of PD‐1 inhibitor plus antiangiogenic agent reported in clinical trials.^14^, ^23^, ^24^, ^25^ Furthermore, our cohort showed a median PFS and median OS of 7.3 months and 19.6 months, respectively. Consistent with our findings, the median PFS and median OS ranged from 4.6 months to 9.3 months, 20.1 months to 26.5 months in the perspective clinical studies, respectively.^14^, ^23^, ^24^, ^25^ Unresectable HCC patients with ECOG PS 2, and a history of prior immunotherapy or antiangiogenic therapy were generally excluded in clinical trials of the combination of PD‐1 inhibitor and antiangiogenic agent,^14^, ^23^ while these patients were enrolled in our real‐world retrospective cohort study. Although the enrollment criteria differed between our study and clinical trials, the efficacy and survival time were similar, verifying PD‐1 inhibitor combined with antiangiogenic therapy was an efficacious strategy for real‐world patients with unresectable HCC.

The novel patterns of response for anti‐PD‐1/PD‐L1 antibody therapy are pseudoprogression (PSPD) and hyperprogressive disease (HPD).^26^ The prevalence of PSPD and HPD across solid tumors is reported below 10% and 4–29%, respectively.^26^, ^27^ Limited literature is available regarding PSPD and HPD in HCC.^28^, ^29^ In our series, seven patients (5.8%) experienced an initial progressive disease defined by the visualization of new lesions and/or increased target lesions, but exacerbation of clinical signs or symptoms was not observed, thus these patients received a confirmation radiological assessment 8 weeks later, six of them was confirmed PD with the second CT scan, while one of them determined to have PD until the third CT scan. Therefore, the incidence of PSPD is rare for HCC patients treated with PD‐1 blockade‐based combination therapies in our study. No HPD was observed in this study. According to the literature, immunotherapy alone rarely leads to HPD,^26^ so as the combination therapy with PD‐1 inhibitor confirmed here.

Currently, different combination patterns of PD‐1/PD‐L1 inhibitor plus antiangiogenic agent are under investigation in clinical trials.^14^, ^23^, ^24^ The combination therapy of sintilimab and VEGF antibody IB305 was adopted as systemic first‐line for advanced HCC patients in China based on phase 2–3 ORIENT‐32 study.^23^ Our study showed that only the type of antiangiogenic agent was associated with tumor response, whether targeting different tyrosine kinases is associated with the efficacy of the combination therapy needs further exploration. Besides, studies have shown that patients with cancer receiving chemotherapy at reduced RDI had worse survival.^30^ However, the impact of RDI on patient receiving antiangiogenic agents remains unclear. RDI of each antiangiogenic agent in our cohort range from 90.67% to 100%, and was not associated with patient survival. Considering the small sample size in some subgroups (e.g., three in bevacizumab group and three in tislelizumab group), further large‐scale clinical trials are needed to verify the efficacy of fixed combination strategy in advanced HCC.

LRTs are commonly used in HCC and have been proved to increase antitumor immune response by exposing neo‐tumor‐associated antigens via tumor necrosis.^31^, ^32^ Currently, the phase 3 LEAP‐012 study is underway evaluating the combination of TACE plus pembrolizumab and lenvatinib in patients with incurable and non‐metastatic HCC.^33^ Additionally, few retrospective studies explored the efficacy and safety of TACE combined with TKI and PD‐1 inhibitor in advanced HCC.^34^, ^35^ In our study, the ORR and median PFS in LRTs group were comparable to those in non‐LRTs group, while patients in LRTs group had a longer OS than those in non‐LRTs group, but had no significant differences. Further randomized control studies are needed.

At present, predictors that enrich for a population more likely to benefit from PD‐1 inhibitor combined with antiangiogenic agent have not been validated in HCC. Our study demonstrated ECOG PS was independent predictor for response and prognosis, while factors as peritoneum metastasis, the presence of previous immunotherapy, Child‐Pugh stage B and high AFP concentration were negative predictors for prognosis in HCC. Clinical trial data and meta‐analyzed real‐world data suggested patients with impaired ECOG PS achieved a lower response rate when treated with PD‐1 inhibitor when compared with good ECOG PS population in a wide range of tumors such as non‐small cell lung cancer, advanced melanoma, and urologic cancer.^36^, ^37^ Similarly, clinical response was related with ECOG PS status in our cohort. ECOG PS has been reported as an independent predictor of survival to immunotherapy in melanoma and non‐small cell lung cancer,^38^, ^39^ consistently, we also observed short PFS and OS in poor ECOG PS group. It is reported tumor response to PD‐1 inhibitors varied among different organs in HCC,^17^ while we found peritoneum metastasis is associated with survival rather than response. Peritoneum is a rare metastatic lesion in advanced HCC, the immune microenvironment in peritoneum is currently unclear and more researches are warranted. AFP is an oncofetal antigen that positively correlated with impaired immune‐stimulatory effect of dendritic cell on T cells,^32^ which might be the underlying mechanism for its negative predictor of prognosis in HCC. To data, the efficacy of safety of immune checkpoint inhibitor (ICI) rechallenge across solid tumors remain unclear, clinical data are limited and data published in the literature are controversial.^40^, ^41^, ^42^ In our study, 14 HCC patients who were previously treated and progressed on anti‐PD‐1 or anti‐PD‐L1 antibodies retreated with the same or different PD‐1 inhibitors (Table S6). Our cohort presented that the history of immunotherapy was a negative predictor of PFS, the possible mechanisms include epigenetics changes, signaling pathway changes, and other checkpoints (e.g., T‐cell immunoglobulin and mucin domain 3 (TIM3) and cytotoxic T‐lymphocyte‐associated protein 4 (CTLA‐4)) might be upregulated.^40^ Current available data are not sufficient to give us clear conclusion and further research is still needed.

The spectrums of TRAEs in our series were consistent with the known AEs of each drug. Grade ≥3 TRAEs to the combination of PD‐1/PD‐L1 inhibitor and antiangiogenic agent occurred from 29% to 93% in clinical trials,^14^, ^15^, ^23^, ^24^, ^43^ higher than those reported in our study (27.9%). Hyperbilirubinemia and thrombocytopenia are known adverse reactions to anti‐PD‐1/PD‐L1 therapy, bleeding is a known AE to antiangiogenic therapy, and patients with cirrhosis and HCC usually have complications of liver insufficiency, thrombocytopenia, and upper gastrointestinal bleeding. Therefore, a comprehensive review and assessment before enrollment to ensure safety is indispensable for patients treated with the combination therapy.

Our study has some limitations. First, it is a retrospective study and the sample size was relatively small, which might reduce the statistic power. Second, the regimens of PD‐1 inhibitors and antiangiogenic agents were heterogeneous and some were off‐label used in the study, though we have analyzed whether the combination strategy would affect the response or prognosis, independent prospective study with fixed regimens need to apply in future. Third, most patients (91.2%) had an etiology of HBV infection rather than other etiological factors in the cohort, thus the results may not be applied for HCC with other etiologies. Finally, biomarkers such as the expression level of PD‐L1 and tumor mutation burden are potential indexes for selecting patients who would benefit from immunotherapy across many cancers, but these biomarkers were not recorded and analyzed in our series.

## 5 CONCLUSION

In summary, we elucidated the efficacy and safety of the combination of PD‐1 inhibitor and antiangiogenic therapy in a real‐world cohort of patients with unresectable HCC, and we identified potential predictors for response and prognosis in HCC, aiming at selecting patients mostly likely to benefit from the combination therapy. Further prospective studies with large‐scale samples, fixed combination strategy, and biomarker detections are needed.

## CONFLICT OF INTEREST

The authors declared no potential conflict of interest.

## AUTHOR CONTRIBUTIONS

Junlin Yao: Conceptualization, data collection, data analysis, and article writing. Xudong Zhu: Data collection, data analysis, and article writing. Zhiheng Wu: Data collection, data analysis, and article writing. Qing Wei: Data collection and review of the manuscript. Yibo Cai: Data collection and review of the manuscript. Yu Zheng: Conceptualization and review of the manuscript. Xinyu Hu: Data collection and review of the manuscript. Hong Hu: Conceptualization and review of the manuscript. Xiangyu Zhang: Review of the manuscript. Hongming Pan: Conceptualization and review of the manuscript. Xian Zhong: Conceptualization, data collection, and writing–review and editing. Weidong Han: Conceptualization, supervision, and writing–review and editing.

## ETHICS STATEMENT

The study was conducted according to the guidelines of the Declaration of Helsinki and approved by the Institutional Review Board of Sir Run Run Shaw Hospital, Hangzhou, Zhejiang, China (no. 20210405–36).

## Supporting information


Figure S1

Table S1

## Data Availability

All raw data are made available upon request at hanwd@zju.edu.cn.
