# Supplementary figures and images for: Efficacy and safety of PD‐1 inhibitor combined with antiangiogenic therapy for unresectable hepatocellular carcinoma: A multicenter retrospective study
Source: Cancer Med. 2022 Apr 10;11(19):3612–22. doi: 10.1002/cam4.4747 (PMC9554456; doi:10.1002/cam4.4747)

Figure S1

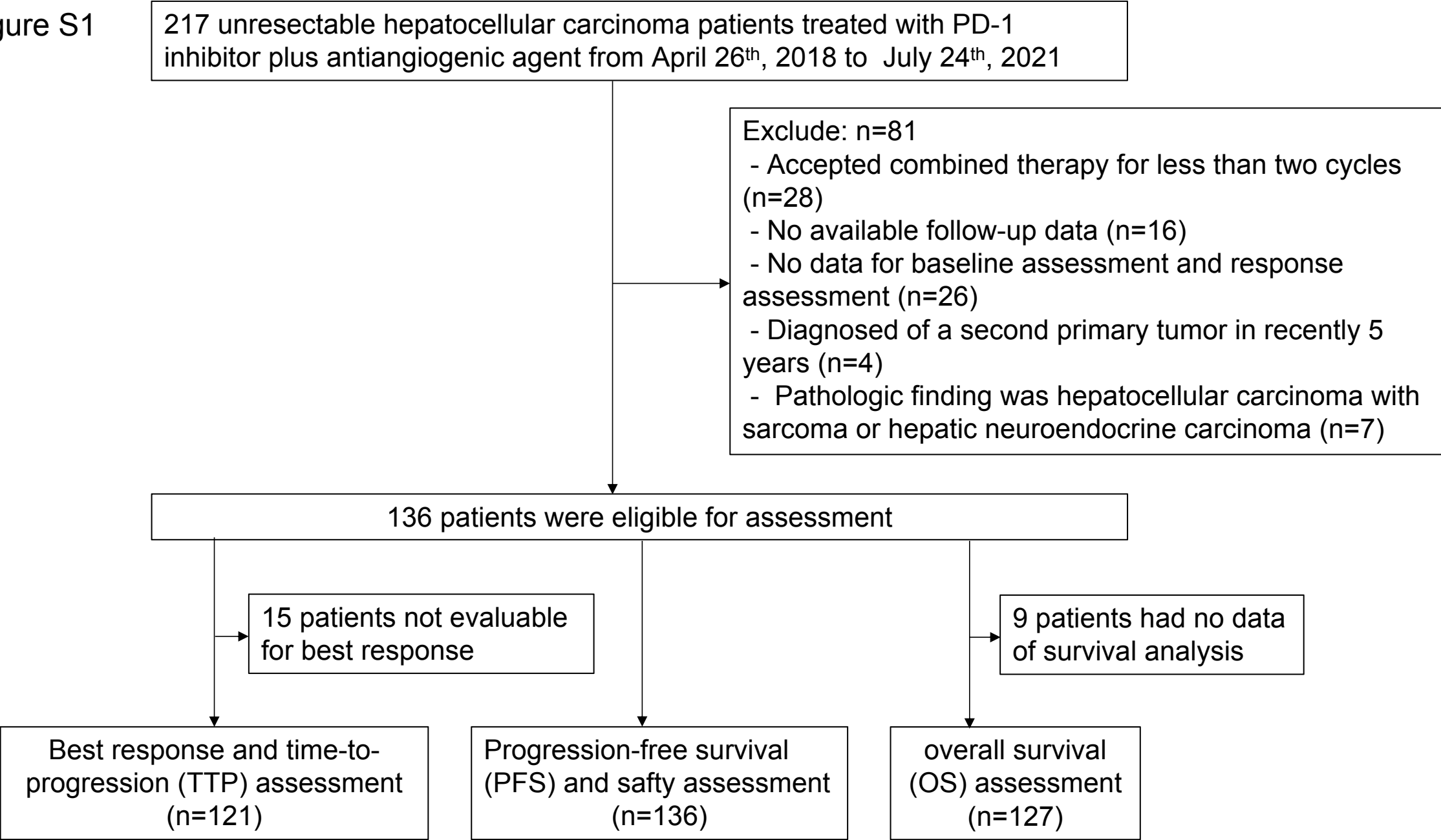

Figure S2

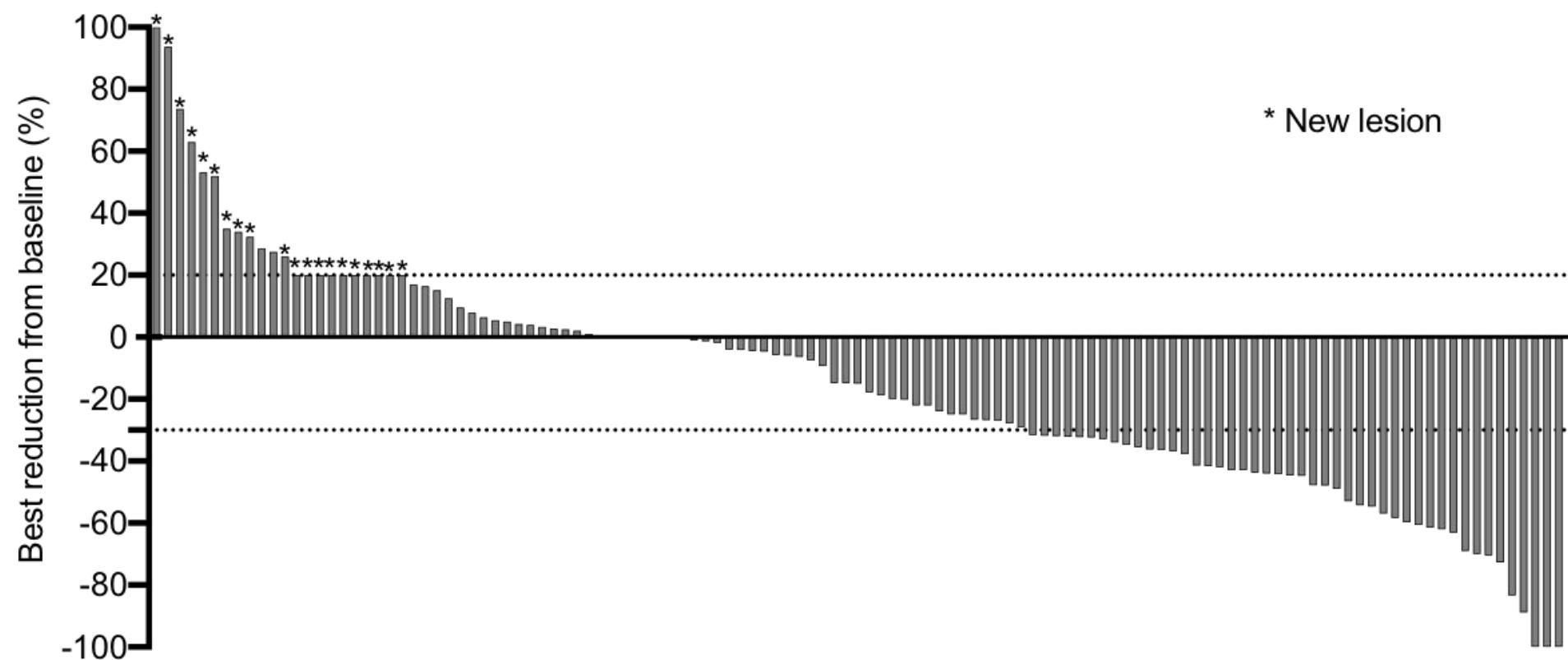

Supplement: Supplementary file 1 — Figure S1 [file CAM4-11-3612-s001.pdf]
