# Supplementary material for: Efficacy and safety of PD‐1 inhibitor combined with antiangiogenic therapy for unresectable hepatocellular carcinoma: A multicenter retrospective study
Source: Cancer Med. 2022 Apr 10;11(19):3612–22. doi: 10.1002/cam4.4747 (PMC9554456; doi:10.1002/cam4.4747)
Supplement: Supplementary file 2 — Table S1 [file CAM4-11-3612-s002.docx]

**Table S1** The therapy strategies of immunotherapy-antiangiogenesis

| Therapy strategies | n (%) |
| --- | --- |
| PD-1 inhibitor | 136 (100) |
| Sintilimab | 57 (41.9) |
| Toripalimab | 34 (25.0) |
| Camrelizumab | 30 (22.1) |
| Pembrolizumab | 7 (5.1) |
| Nivolumab | 5 (3.7) |
| Tislelizumab | 3 (2.2) |
| Antiangiogenic drug | 136 (100) |
| Lenvatinib | 57 (41.9) |
| Sorafenib | 45 (33.1) |
| Regorafenib | 16 (11.8) |
| Apatinib | 15 (11.0) |
| Bevacizumab | 3 (2.2) |

Abbreviations: PD-1, programmed death 1.

**Table S2** The therapy strategies of additional LRT

|  | n (%) | Frequency  (mean ± SD) |
| --- | --- | --- |
| LRT | 63 (46.3) | 1.8 ± 1.1 |
| TACE only | 34 (25.0) | 1.5 ± 0.8 |
| Ablation only | 4 (2.9) | 1.5 ± 1.0 |
| Radiation only | 6 (4.4) | 1.0 ± 0 |
| Seed implantation only | 3 (2.2) | 1.0 ± 0 |
| At least two forms of locoregional therapy | 16 (11.8) | 2.9 ± 1.1 |
| TACE plus seed implantation | 9 (6.6) | 3.1 ± 1.1 |
| TACE plus radiation | 7 (5.1) | 2.7 ± 1.1 |

Abbreviations: LRT, locoregional therapy; TACE, transcatheter arterial chemoembolization.

**Table S3** The RDI of antiangiogenic agents

| Antiangiogenic drug | n (%) | RDI (mean ± SD) |
| --- | --- | --- |
| Total | 136 (100) | 97.24 ± 7.79 |
| Lenvatinib | 57 (41.9) | 97.37 ± 7.97 |
| Sorafenib | 45 (33.1) | 98.11 ± 6.33 |
| Regorafenib | 16 (11.8) | 100 |
| Apatinib | 15 (11.0) | 90.67 ± 12.08 |
| Bevacizumab | 3 (2.2) | 100 |

Abbreviations: RDI, relative dose intensity. SD, standard deviation.

**Table S4** The baseline characteristics of antiangiogenic agents

| Characteristics and therapeutic strategies | All patients  n=136 | Lenvatinib  n=57 | Sorafenib  n=45 | Regorafenib  n=16 | Apatinib  n=15 | Bevacizumab  n=3 |
| --- | --- | --- | --- | --- | --- | --- |
| Ages (years) |  |  |  |  |  |  |
| Median (range) | 58 (14-84) | 56 (14-84) | 61 (31-81) | 60.5 (47-79) | 57 (47-69) | 58 (55-77) |
| ≥ 60 | 62 (45.6) | 23 (40.4) | 23 (51.1) | 8 (50.0) | 7 (46.7) | 1 (33.3) |
| Sex |  |  |  |  |  |  |
| Male | 115 (84.6) | 46 (80.7) | 40 (88.9) | 14 (87.5) | 13 (86.7) | 2 (66.7) |
| Female | 21 (15.4) | 11 (19.3) | 5 (11.1) | 2 (12.5) | 2 (13.3) | 1 (33.3) |
| ECOG performance status |  |  |  |  |  |  |
| 0-1 | 78 (57.4) | 34 (59.6) | 20 (44.4) | 8 (50.0) | 13 (86.7) | 3 (100.0) |
| 2 | 58 (42.6) | 23 (40.4) | 25 (55.6) | 8 (50.0) | 2 (13.3) | 0 |
| Alcohol use |  |  |  |  |  |  |
| Current or previous | 54 (39.7) | 25 (43.9) | 16 (35.6) | 7 (43.8) | 5 (33.3) | 1 (33.3) |
| Never | 82 (60.3) | 32 (56.1) | 29 (64.4) | 9 (56.3) | 10 (66.7) | 2 (66.7) |
| Metastasis present |  |  |  |  |  |  |
| Extrahepatic disease | 101 (74.3) | 46 (80.7) | 27 (60.0) | 13 (81.3) | 13 (86.7) | 2 (66.7) |
| Lung | 52 (38.2) | 20 (35.1) | 15 (33.3) | 8 (50.0) | 8 (53.3) | 1 (33.3) |
| Lymph nodes | 61 (44.9) | 32 (56.1) | 16 (35.6) | 5 (31.3) | 6 (40.0) | 2 (66.7) |
| Bone | 11 (8.1) | 5 (8.8) | 2 (4.4) | 4 (25.0) | 0 | 0 |
| Peritoneum | 12 (8.8) | 5 (8.8) | 1 (2.2) | 3 (18.8) | 2 (13.3) | 1 (33.3) |
| Intra-abdominal implantation | 7 (5.1) | 4 (7.0) | 0 | 1 (6.3) | 1 (6.7) | 1 (33.3) |
| Adrenal gland | 7 (5.1) | 3 (5.3) | 1 (2.2) | 1 (6.3) | 2 (13.3) | 0 |
| Child-Pugh stage |  |  |  |  |  |  |
| A | 109 (80.1) | 45 (78.9) | 35 (77.8) | 12 (75.0) | 14 (93.3) | 3 (100.0) |
| B | 27 (19.9) | 12 (21.1) | 10 (22.2) | 4 (25.0) | 1 (6.7) | 0 |
| BCLC stage |  |  |  |  |  |  |
| B | 12 (8.8) | 4 (7.0) | 5 (11.1) | 1 (6.3) | 1 (6.7) | 1 (33.3) |
| C | 124 (91.2) | 53 (93.0) | 40 (88.9) | 15 (93.8) | 14 (93.3) | 2 (66.7) |
| Alpha-Fetoprotein |  |  |  |  |  |  |
| < 400 (IU/ml) | 73 (53.7) | 24 (42.1) | 28 (62.2) | 13 (81.3) | 7 (46.7) | 1 (33.3) |
| ≥ 400 (IU/ml) | 63 (46.3) | 33 (57.9) | 17 (37.8) | 3 (18.8) | 8 (53.3) | 2 (66.7) |
| Macrovascular invasion | 68 (50.0) | 34 (59.6) | 20 (44.4) | 9 (56.3) | 5 (33.3) | 0 |
| Viral status |  |  |  |  |  |  |
| Uninfected | 12 (8.8) | 3 (5.3) | 3 (66.7) | 4 (25.0) | 2 (13.3) | 0 |
| Hepatitis B | 124 (91.2) | 54 (94.7) | 42 (93.3) | 12 (75.0) | 13 (86.7) | 3 (100.0) |
| Hepatitis C | 0 | 0 | 0 | 0 | 0 | 0 |
| Liver cirrhosis | 108 (79.4) | 46 (80.7) | 38 (84.4) | 12 (75.0) | 10 (66.7) | 2 (66.7) |
| Prior therapies |  |  |  |  |  |  |
| Surgery | 67 (49.3) | 24 (42.1) | 19 (42.2) | 12 (75.0) | 10 (66.7) | 2 (66.7) |
| LRT^a^ | 92 (67.6) | 37 (64.9) | 27 (60.0) | 15 (93.8) | 10 (66.7) | 3 (100.0) |
| Immunotherapy | 14 (10.3) | 5 (8.8) | 5 (11.1) | 2 (12.5) | 1 (6.7) | 1 (33.3) |
| Antiangiogenic therapy | 47 (34.6) | 20 (35.1) | 8 (17.8) | 14 (87.5) | 4 (26.7) | 1 (33.3) |
| Previous systemic treatment line |  |  |  |  |  |  |
| 0 | 80 (58.8) | 35 (61.4) | 33 (73.3) | 0 | 11 (73.3) | 1 (33.3) |
| ≥1 | 56 (41.2) | 22 (38.6) | 12 (26.7) | 16 (100.0) | 4 (26.7) | 2 (66.7) |
| with additional LRT^a^ | 63 (46.3) | 28 (49.1) | 26 (57.8) | 6 (37.5) | 2 (13.3) | 1 (33.3) |

Note: data presented as n (%)

Abbreviations: BCLC, Barcelona Clinic Liver Cancer; ECOG, Eastern Cooperative Oncology Group; LRT, locoregional therapy; TACE, transcatheter arterial chemoembolization.

^a^LRT includes TACE, ablation, radiation or seed implantation.

**Table S5** The influence of therapy strategies on treatment responses, PFS and OS

| Therapy strategies | Response | | PFS | | OS | |
| --- | --- | --- | --- | --- | --- | --- |
|  | OR (95% CI) | *P* value | HR (95% CI) | *P* value | HR (95% CI) | *P* value |
| PD-1 inhibitor |  | .356 |  | .710 |  | .897 |
| Sintilimab | 1 (Ref) |  | 1 (Ref) |  | 1 (Ref) |  |
| Toripalimab | 1.0 (0.4-2.5) | .982 | 0.8 (0.5-1.2) | .229 | 0.8 (0.4-1.7) | .622 |
| Camrelizumab | 1.3 (0.5-3.7) | .573 | 0.8 (0.5-1.2) | .235 | 0.9 (0.5-1.9) | .845 |
| Pembrolizumab | 0.9 (0.1-5.8) | .904 | 1.0 (0.4-2.2) | .950 | 0.9 (0.3-3.1) | .908 |
| Nivolumab | 0.6 (0.1-4.6) | .617 | 1.0 (0.4-2.4) | .910 | 1.2 (0.4-4.1) | .726 |
| Tislelizumab | NA | .999 | 0.5 (0.1-2.0) | .329 | NA | .975 |
| Antiangiogenic drug |  | .029 |  | .534 |  | .182 |
| Lenvatinib | 1 (Ref) |  | 1 (Ref) |  | 1 (Ref) |  |
| Sorafenib | 1.9 (0.8-4.5) | .138 | 0.9 (0.6-1.4) | .672 | 0.4 (0.2-1.9) | .422 |
| Regorafenib | 6.4 (1.3-31.5) | .021 | 1.3 (0.7-2.4) | .331 | 0.7 (0.3-1.7) | .456 |
| Apatinib | 1.3 (0.4-4.6) | .698 | 0.8 (0.4-1.4) | .406 | 0.7 (0.3-1.7) | .468 |
| Bevacizumab | NA | .999 | 0.5 (0.1-2.1) | .364 | 0.9 (0.2-3.0) | .929 |
| With additional LRT |  | .309 |  | .680 |  | .078 |
| Yes | 1 (Ref) |  | 1 (Ref) |  | 1 (Ref) |  |
| No | 0.7 (0.3-1.4) | .309 | 1.1 (0.7-1.6) | .680 | 0.6 (0.4-1.0) | .078 |

Abbreviations: CI, confidence interval; HR, hazard ratio; LRT, locoregional therapy; NA, not applicable; OR, odds ratio; OS, overall survival; PD-1, programmed death 1; PFS, progression-free survival; Ref, reference.

**Table S6** Detail data on ICI rechallenge in unresectable HCC patients

| Patient no. | History of immunotherapy | | | | Current immunotherapy-antiangiogenesis | | |
| --- | --- | --- | --- | --- | --- | --- | --- |
|  | PD-1/PD-L1 inhibitor | Combined strategy | Best response | PFS (m) | PD-1 inhibitor | Best response | PFS (m) |
| 1 | IBI308-from clinical trial | PolyI:C-from clinical trial | PR | 10.20 | Sintilimab | PR | 12.50 |
| 2 | Sintilimab | No combination | PR | 4.61 | Sintilimab | CR | 14.90 |
| 3 | Atezolizumab-  from clinical trial | Bevacizumab-  from clinical trial | PR | 11.3 | Camrelizumab | PD | 2.07 |
| 4 | Cadonilimab-from clinical trial | Anti-CTLA-4 antibody-from clinical trial | SD | 3.90 | Camrelizumab | NA | 12.73 |
| 5 | Camrelizumab | No combination | SD | 4.13 | Sintilimab | PD | 1.73 |
| 6 | Sintilimab | Chemotherapy | SD | 2.94 | Sintilimab | PD | 1.17 |
| 7 | Toripalimab | No combination | SD | 3.83 | Toripalimab | SD | 4.17 |
| 8 | Sintilimab | No combination | PD | 2.03 | Sintilimab | SD | 3.57 |
| 9 | Sintilimab | No combination | PD | 1.17 | Sintilimab | PR | 3.93 |
| 10 | IBI308-from clinical trial | PolyI:C-from clinical trial | PD | 2.83 | Sintilimab | SD | 5.10 |
| 11 | Sintilimab | No combination | PD | 1.67 | Sintilimab | PD | 2.27 |
| 12 | Toripalimab | No combination | PD | 2.57 | Sintilimab | NA | 5.47 |
| 13 | Camrelizumab | No combination | PD | 1.37 | Camrelizumab | SD | 4.43 |
| 14 | Pembrolizumab-  from clinical trial | Lenvatinib-  from clinical trial | PD | 2.53 | Nivolumab | SD | 2.03 |

Abbreviations: CR, complete response; CTLA-4; cytotoxic T-lymphocyte–associated protein 4; NA, not available; no., number; PD, progressive disease; PD-1, programmed death 1; PD-L1, programmed death ligand 1; PFS, progression-free survival; PolyI:C, polyinosinic acid-polycytidylic acid; PR, partial response; SD stable disease.
